# Enhancing rumen microbial diversity and its impact on energy and protein metabolism in forage-fed goats

**DOI:** 10.3389/fvets.2023.1272835

**Published:** 2023-12-21

**Authors:** Alejandro Belanche, Juan Manuel Palma-Hidalgo, Elisabeth Jiménez, David R. Yáñez-Ruiz

**Affiliations:** ^1^Estación Experimental del Zaidín (CSIC), Granada, Spain; ^2^Department of Animal Production and Food Sciences, University of Zaragoza, Zaragoza, Spain

**Keywords:** energy metabolism, forage digestion, multi-kingdom, protein metabolism, protozoa, rumen microbiota, ruminants

## Abstract

**Introduction:**

This study explores if promoting a complex rumen microbiota represents an advantage or a handicap in the current dairy production systems in which ruminants are artificially reared in absence of contact with adult animals and fed preserved monophyte forage.

**Methods:**

In order to promote a different rumen microbial diversity, a total of 36 newborn goat kids were artificially reared, divided in 4 groups and daily inoculated during 10 weeks with autoclaved rumen fluid (AUT), fresh rumen fluid from adult goats adapted to forage (RFF) or concentrate (RFC) diets, or absence of inoculation (CTL). At 6 months of age all animals were shifted to an oats hay diet to determine their ability to digest a low quality forage.

**Results and discussion:**

Early life inoculation with fresh rumen fluid promoted an increase in the rumen overall microbial diversity which was detected later in life. As a result, at 6 months of age RFF and RFC animals had higher bacterial (+50 OTUs) and methanogens diversity (+4 OTUs) and the presence of a complex rumen protozoal community (+32 OTUs), whereas CTL animals remained protozoa-free. This superior rumen diversity and presence of rumen protozoa had beneficial effects on the energy metabolism allowing a faster adaptation to the forage diet, a higher forage digestion (+21% NDF digestibility) and an energetically favourable shift of the rumen fermentation pattern from acetate to butyrate (+92%) and propionate (+19%) production. These effects were associated with the presence of certain rumen bacterial taxa and a diverse protozoal community. On the contrary, the presence of rumen protozoa (mostly *Entodinium*) had a negative impact on the N metabolism leading to a higher bacterial protein breakdown in the rumen and lower microbial protein flow to the host based on purine derivatives urinary excretion (-17% to -54%). The inoculation with autoclaved rumen fluid, as source of fermentation products but not viable microbes, had smaller effects than using fresh inoculum. These findings suggest that enhancing rumen microbial diversity represents a desirable attribute when ruminants are fed forages in which the N supply does not represent a limiting factor for the rumen microbiota.

## Introduction

Evolution has allowed ruminants to develop a complex, multi-chambered forestomach and a system of regurgitation and rumination in order to establish an enhanced rumen microbial fermentation. This anaerobic fermentation is conducted by rumen bacteria, methanogenic archaea, anaerobic fungi, protozoa and phages and provides several competitive advantages but also some drawbacks. Increased rumen microbial protein synthesis and fiber digestion are thought to represent important overall evolutionary advantages for wild ruminants grazing (or browsing) highly diverse forages and bushes (1). However, in modern ruminant production systems ruminants are often fed elevated proportions of highly fermentable feeds in which the concentrate can represents up to 55, 85, and 92% of the total diet for dairy cows, dairy goats and feedlot systems, respectively (2). This type of diets often leads to digestive disorders such as rumen acidosis and diarrhea which often require the use of feed additives to maintain productivity and health (3). On the contrary, in meat-orientated systems or low nutrient requiring situations, ruminants are often fed low quality diets with a high proportion of forage (up to 100%). Additionally, there is an increasing trend to feed ruminants with preserved forages (i.e., hay and silage) which are often made with a single botanical species, aspect that represents an over-simplification of the diets which ruminants were originally developed for.

In relation to the rearing system, it has been demonstrated that a progressive rumen microbial colonization and functional development occur when young ruminants are reared with the dam or adult companions, allowing a natural rumen microbial transfer to the offspring (4) and feeding behavior learned from the adults (5) resulting on a superior forage digestion and animal growth than artificially reared ruminants (6). On the contrary, in modern intensive dairy systems, the newborns are usually separated from their dams after birth and fed milk replacer or whole milk. This absence of contact with adult animals often leads to a delay in the rumen microbial and physiological development that can persist as long as the animals are not in contact with adult ruminants (7, 8). In the last decades, important efforts have been made to optimize the artificial rearing of young ruminants such as improvements of the colostrum feeding, the development of high-quality liquid feed, texturized starter feeds, feed additives with biologically active substances or the implementation of high quality forages (9). This has allowed to optimize the anatomical and physiological development of the rumen mucosa and associated papillae which is key for a successful post-weaning process (10). Moreover, several authors have evaluated the effects of inoculating young ruminants with fresh (11–13) or lyophilized rumen fluid from adult ruminants (14, 15). Most of these studies have reported an acceleration of the rumen microbial and functional development. In a previous publication related to the present experiment, it was noted that the inoculation with fresh rumen fluid had positive effects during the weaning process (16), however, the persistency of these effects later in life and the potential effects on the productive performances remain unknown.

The objectives of this study were to investigate the long-term effects of enhancing the rumen microbial diversity in forage-fed goats by early-life inoculation with rumen fluid from adult ruminants. A multi-kingdom meta-taxonomic community analysis including bacteria, methanogens, protozoa and anaerobic fungi was performed to have a detailed description for the rumen microbiome, and to identify the key microbes associated with changes in the rumen fermentation, feed utilization and productive performance.

## Materials and methods

### Inocula preparation

This study involved the inoculation of young goat kids with various types of rumen inocula obtained from adult goats to enhance rumen microbial diversity. A comprehensive description of the inocula preparation process has been published in previous studies (13, 16). Briefly, four adult goats, each equipped with a permanent rumen fistula, were fed two different diets. Four of them were fed a forage diet consisting of oats hay, while the remaining four received a high-concentrate diet (75% concentrate feed and 25% oats hay) to generate distinct rumen microbial inocula. After 2 weeks of adaptation to the diet, rumen fluid from the donor goats fed forage (RFF) or concentrate diet (RFC) were collected daily 2 h after the morning feeding, pooled by diet, strained through a cheesecloth, maintained in anaerobic conditions in a thermal flask and orally inoculated as fresh inoculum to young goat kids. Additionally, autoclaved inoculum (AUT) was generated weekly by combining equal volumes of RFF and RFC inocula. This mixture was autoclaved at 115°C for 30 min to lyse all microbes while preserving the rumen fermentation products. Samples from each type of inocula were collected for inocula characterization.

### Inoculation

**Figure 1:**
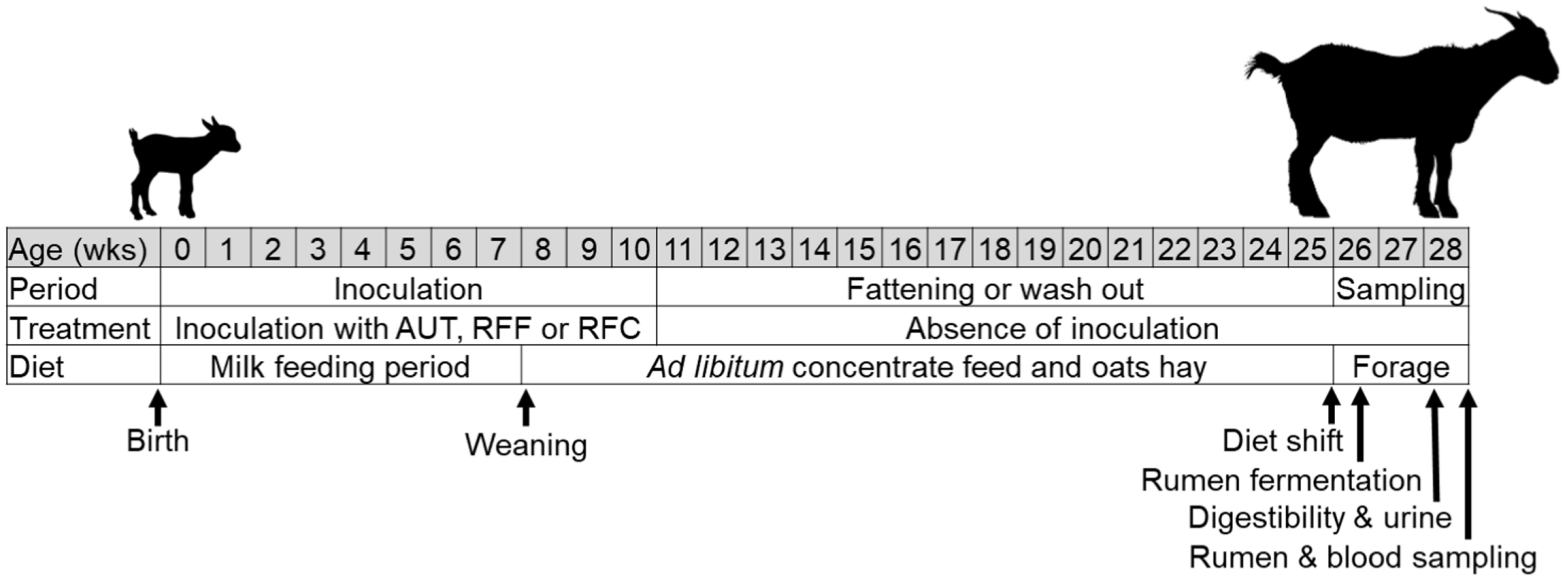
Ilustration of the diferent stages of the experimental set up. Animals received an daily and oral inoculation with autoclaved rumen fluid (AUT), rumen fluids from adult animals fed forage (RFF), or fed concentrate diets (RFC) or without inoculation (CTL).

A total of 36 newborn Murciano-Granadina goat kids were nourished with natural colostrum as previously described (16). These kids were randomly allocated into 4 experimental treatments (*n* = 9) which were kept physically separated during the entire experiment. The experiment consisted in three periods (Figure 1): (1) the inoculation period (from birth to week 10 of age) to promote a different degree of rumen microbial development, (2) the fattening or wash out period (from week 10 to 25 of age), and (3) the sampling period after the animals were shift to a full forage diet (from week 26–28 of age).

Experimental treatments involved oral and daily inoculation (2.5 mL/animal during week 1 and 5 mL/animal thereafter) with AUT, RFF, RFC or absence of inoculation (CTL). All goat kids were fed a commercial milk replacer (Univet Spray, Cargill, Barcelona, Spain, declared composition in DM: 92.8% OM, 24% CP and 22% EE) which was freshly prepared by mixing with warm water (170 g/L) twice per day (at 09:00 and 17:00 h) and offered *ad libitum*. From 2 weeks of age, all animals had *ad libitum* access to a pelleted concentrate (0–14 Rumiantes Transición, Macob, Granada, Spain, chemical composition in g/kg DM, OM 938, CP 199, NDF 323, ADF 132, ADL 15) and oats hay (OM 930, CP 79, NDF 634, ADF 280, ADL 55). At 7 weeks of age, all animals were weaned by gradually decreasing the solids concentration of milk replacer during 4 consecutive days (−20, −40%, −60 and −80%, respectively). During the fattening or wash out period the animals remained physically separated in four groups and were fed the same concentrate feed and oats hay as described before (both *ad libitum*), but they did not receive any further inoculation. This allowed to assess the persistency of the treatment effects later in life. Finally, at 26 weeks of age, an abrupt dietary shift was implemented, involving the removal of access to concentrate feed while providing access to forage (oats hay) as the sole dietary ingredient. This abrupt change in diet served as a means to assess the animals’ ability to adapt to a forage diet.

### Rumen and blood sampling

Animals were housed in individual pens (2 × 2 m) and daily feed intake and BW change was monitored during 21 days after the dietary change. Rumen fermentation was evaluated at 4 and 21 d after this dietary shift. Rumen content (approximately 50 mL) was withdrawn for each animal by oro-gastric intubation at 0900 h as previously described (17). Rumen samples were filtered through a cheesecloth and solids discarded given their small and variable proportion in the samples. Then, pH was immediately measured and 3 subsamples were taken for volatile fatty acids (VFA), ammonia and lactate determinations, as previously described (18). Rumen VFA concentrations were determined by a GC system coupled with a Flame Ionization Detector (Auto-System, Perkin Elmer, Waltham, MA) whereas ammonia (19) and lactate concentrations (20) were measured using a colorimetric methods. Moreover, two additional sub-samples were collected on day 21, one was snap frozen in liquid N to describe the rumen microbiota, while the other sample (12.5 mL) was mixed with 37.5 mL of anaerobic buffer and incubated at 39°C for 24 h in 120-ml Wheaton bottles to determine gas and CH_4_ production *in vitro*. Blood samples (4 mL) were collected at 0900 h from the jugular vein (on day 21), placed in tubes without anticoagulant, centrifuged at 2,000 x *g* for 15 min. Serum samples were frozen and sent to the “Laboratorio de Técnicas Instrumentales” from University of Leon (Spain) to determine concentrations of glucose, β-hydroxybutyrate, blood pH, pCO_2_, tCO_2_, Na^+^, K^+^, HCO3^−^, Cl^−^ and anion gap using an auto-analyzer (BA400, Bio-Systems, Barcelona, Spain). To assess the potential effects of the experimental treatments on the stress levels induced by the dietary shift, cortisol concentration in hair was measured as previously reported (16). Briefly, a surface of 25 cm^2^ in the dorsal neck was shaved before (day 0) and after 21d the dietary shift and cortisol concentration was measured using a commercial kit (Cortisol ELISA Saliva, ALPCO, Salem, NH).

### Digestibility and microbial protein synthesis

After 17d of adaptation to the diet, goats were placed in metabolic crates to determine feed digestibility during 4 consecutive days. Due to the limited number of metabolic crates, goats were divided into two periods with equal number of goats per treatment within each period. Feed intake and fecal excretion were daily monitored and feed refusals were sampled, pooled per animal and analyzed to calculate the feed nutrients intake and digestibility. Water consumption was recorded and urine excretion was collected in buckets containing 50 mL of H_2_SO_4_ (10% vol/vol). Aliquots representing 10% of the daily fecal and urinary production were pooled and stored at −20°C for further analyses.

### Chemical composition

The starter concentrate used in this study was freshly made in a commercial mill (Cereales Macob, Granada, Spain) and consumed before the expiration date to prevent the potential presence of mycotoxins and its negative effects on productivity and health (21). Dry matter (DM) and organic matter (OM) concentrations were determined using the method 934.01, 942.05, respectively (22). Nitrogen (N) content was also measured in solid (method 990.03) and urine samples (method 993.13). Neutral detergent fiber (NDF) and acid detergent fiber (ADF) were measured (23) using an Ankom 220 fiber analyzer unit (Ankom Technology Crop., Macedon, NY) with α-amylase and expressed without residual ash. The concentration of purine derivatives (PD) and creatinine in urine samples were determined using a HPLC system (24) using allopurinol as an internal standard.

### Rumen microbiota

For rumen microbial characterization frozen samples were freeze-dried, physically disrupted by bead-beating for 1 min and DNA was extracted using a commercial kit (QIAamp DNA Stool Mini Kit, Qiagen LTd., Barcelona, Spain). Rumen concentration of the different microbial groups were determined by qPCR using serial dilutions of microbial standards (6) and specific primers for the 16S rRNA gene for bacteria, the mcrA gene for methanogens and 18S rRNA genes for protozoal and anaerobic fungi (Supplementary Table S1).

For rumen meta-taxonomic analyses, DNA samples were sent to University of Illinois Biotechnology Center (Urbana, IL, USA) for amplicon sequencing using Miseq V3 (Ilumina Inc., San Diego, CA, USA) as previously described (13). Briefly, specific primers sets were used to amplify bacterial 16S (V3-V5 region), methanogens 16S (V3-V4 region), protozoal 18S (V4-V6 region) and anaerobic fungi ITS3-ITS4 regions (Supplementary Table S1). For each of the 4 major microbial groups, samples were primer-sorted, demultiplexed and paired-end reads were merged into one file. Downstream analysis was performed using QIIME2 (Version 2021.4) for bacteria and methanogens (25), PIPITS for fungi (26) and IM-Tornado for protozoa (27). Low-quality reads (<Q25) were trimmed and chimeras were removed using chimera.vsearch (28). All sequences were grouped into operational taxonomic units (OTUs) with a similarity cut-off of 97%. The resulting OTUs were taxonomically classified using the Silva_138 database (29) for bacteria and protozoa, RIM-DB for methanogens (30) and UNITE for fungi (31). After taxonomical classification, data from each of the 4 major microbial groups was processed separately. The number of sequences per sample was normalized for each microbial group and singletons were removed. The relative abundance of each OTU was determined along with the Good’s coverage, and alpha diversity.

### Calculations and statistical analyses

Statistical calculations were performed using SPSS software (IBM Corp., Version 21.0, NY, USA). For rumen fermentation data were analyses based on a repeated measures mixed-effects (residual maximum likelihood) as follows:

$Y_{ijkl}=\mu +I_{i}+T_{j}+{\left(I\times T\right)}_{ij}+G_{k}+A{\left(G\right)}_{l}+e_{ijkl}$

Where Y_ijkl_ is the dependent, continuous variable, *μ* is the overall population mean, *I_i_* is the fixed effect fo the inoculation (*I* = CTL vs. AUT vs. RFF vs. RFC), *T_j_* is the fixed effect of the sampling time (*j* = 4d vs. 21d), *(I × T)_ij_* is the interaction term, *G_k_* is the random effect of the period (*k* = 1 vs. 2), *A(G)_l_* is the random effect of the animal nested to the period (*l* = 1–36), and *e_ijkl_* is the residual error. For blood metabolites, digestibility, urinary PD excretion and qPCR only one sample time was considered (21d) and data were analyzed by ANOVA excluding the time as a factor. Fermentable organic matter (FOM) was calculated according to (32). Taxa abundances (in %) were tested for normality using the Shapiro–Wilk test and data were analyzed using the Kruskal-Wallis non-parametric test. False discovery rate was minimized by using the Bonferroni *post hoc* test. Significant effects were declared at *p* < 0.05 and tendency to difference at *p* < 0.1.

Treatment effects on the bacterial, methanogens, protozoal and anaerobic fungi communities were assessed based on the Bray-Curtis distance metrics using the UPGMA function (PRIMER-6 software, PRIMER-E Ltd., Plymouth, UK). Abundances of each OTU were log10-transformed and data were analyzed by PERMANOVA after 999 random permutations of residuals under the reduced model using the Montecarlo test. When significances were detected, pair-wise comparisons were performed across treatments. For each microbial group, a principal Coordinate analysis (PCoA) was conducted to illustrate the impact of the treatments on the overall community structure, and tripod vectors were included to indicate the relationships between the community structure and the metadata consisting in 31 variables including rumen fermentation, digestibility, purine derivatives, blood metabolites and microbial diversity. A multi-kingdom analysis was conducted including rumen abundances from all microbial groups in order to assess the treatments effects on the overall rumen microbiome. Spearman correlations were calculated to identify relationships between the microbial taxa abundances and the metadata but only strong correlations were considered (*ρ* ≥ 0.4 or ≤ −0.4 and *p* < 0.001).

## Results

### Inoculum and rumen fermentation

Fermentative and microbiological differences across rumen fluids used as inoculum has been previously described [Supplementary Table S2; (13)]. All animals required an adaptation process after the abrupt dietary shift from a high-concentrate to a full-forage diet as noted in the rumen fermentation data from 4 and 21 days after the dietary shift (Table 1). This adaptation included an increase in the DMI (*p* = 0.098), total VFA (*p* < 0.001), FOM (*p* < 0.001), butyrate (*p* = 0.042) and iso-acids molar proportions (i.e., iso-butyrate and iso-valerate) and lower rumen ammonia (*p* = 0.042). However, RFF and RFC tended to have a higher DMI (*p* = 0.094) at day 4 after the dietary shift. This inoculation with RFF or RFC inocula also promoted higher rumen ammonia concentration (*p* = 0.003) and proportions of butyrate (*p* < 0.001) and propionate (*p* = 0.023) in detriment to acetate (*p* < 0.001) in comparison to the CTL treatment across both sampling times. Animals inoculated with autoclaved rumen fluid showed intermediate rumen fermentation values between the CTL and the RFF or RFC animals.

**Table 1:** Feed intake and rumen fermentation in 26-week-old goats measured at 4 and 21 days after a shift from a high-concentrate to a full forage diet (oats hay).

		Treatments^1^					*p*-values		
	Day	CTL	AUT	RFF	RFC	SED	Ino.	Time	IxT
DMI, g/d	4d	432^b^	500^ab^	502^a^	516^a^	39.37	0.642	0.098	0.094
	21d	517	535	493	500				
Rumen pH	4d	6.83	6.88	6.68	6.83	0.118	0.164	0.339	0.879
	21d	6.95	6.91	6.76	6.83				
N-NH_3_, mg/dL	4d	1.56^b^	2.61^ab^	4.60^a^	3.16^a^	0.960	0.003	0.042	0.720
	21d	0.42^b^	1.82^a^	2.77^a^	2.75^a^				
Lactate, ng/L	4d	189	180	172	197	18.20	0.188	<0.001	0.198
	21d	66.0	110	93.6	116				
Total VFA, mmol/L	4d	44.7	43.9	54.0	49.9	10.36	0.485	<0.001	0.927
	21d	62.8	71.4	75.1	75.0				
Proportion, %									
Acetate	4d	75.3^a^	70.3^b^	67.5^b^	68.5^b^	1.373	<0.001	0.083	0.279
	21d	74.6^a^	70.3^b^	69.4^b^	70.7^b^				
Propionate	4d	16.2^b^	19.2^b^	20.2^a^	18.4^ab^	1.347	0.023	0.293	0.861
	21d	16.5	18.9	19.2	17.3				
Butyrate	4d	4.97^c^	7.30^b^	9.27^a^	9.87^a^	0.833	<0.001	0.042	0.175
	21d	7.00^c^	8.34^b^	9.07	9.91^ab^				
Isobutyrate	4d	0.84	0.97	1.08	1.07	0.119	0.173	<0.001	0.321
	21d	0.67^b^	0.92^a^	0.79^ab^	0.78^ab^				
Valerate	4d	1.67^a^	1.23^b^	0.99^b^	1.22^b^	0.157	0.009	<0.001	0.190
	21d	0.93	0.79	0.72	0.78				
Isovalerate	4d	1.01	0.99	0.99	0.98	0.194	0.352	0.001	0.291
	21d	0.30^b^	0.74^a^	0.82^a^	0.56^ab^				
FOM, mmol/L	4d	23.9	23.9	29.7	28.0	5.782	0.398	<0.001	0.928
	21d	33.8	38.9	41.0	41.6				

1 During the first 10 weeks of age goats (*N* = 36) received a daily and oral inoculation with autoclaved rumen fluid (AUT), rumen fluid from adult animals fed forage (RFF), or fed concentrate diets (RFC) or without inoculation (CTL). Means within a raw with different superscript differ (*p* < 0.05).

### Blood metabolites, digestibility and microbial protein synthesis

All animals remained in good health and no differences were noted neither on the hair cortisol levels in hair nor in the serum metabolite concentrations (Table 2). No differences between AUT, RFF and RFC were noted in terms of apparent feed digestibility, but these three treatments had substantially higher digestibility values for DM (*p* = 0.014), OM (*p* = 0.007), N (*p* = 0.092), NDF (*p* < 0.001) and ADF (*p* < 0.001) than CTL animals. *In vitro* gas production and CH_4_ production were higher for AUT, RFF and RFC than for the CTL treatment, whereas no differences were noted when CH_4_ emission was normalized for FOM.

**Table 2:** Blood parameters, feed digestibility and microbial protein synthesis in 26-week-old goats measured at 21 days after a shift from a high-concentrate to a full forage diet (oats hay).

Treatments^1^	CTL	AUT	RFF	RFC	SED	*p*-value
BW, kg	24.3	25.1	24.2	23.6	1.383	0.757
Cortisol in hair, ng/mg	1.50	1.53	1.49	1.54	0.144	0.982
Blood metabolites, mmol/L						
Glucose	2.86	3.20	2.99	2.89	0.240	0.482
β-hydroxybutyrate	2.63	2.50	2.84	2.17	0.381	0.327
Blood pH	7.30	7.33	7.35	7.33	0.028	0.222
pCO_2_, mm of Hg	56.0	48.4	46.6	46.8	3.960	0.088
tCO_2_, mm of Hg	27.0	24.8	25.2	24.3	1.299	0.410
Na^+^	145	146	146	144	0.817	0.121
K^+^	4.75	5.03	4.91	4.84	0.174	0.417
HCO_3_^−^	25.3	23.4	23.8	22.8	1.217	0.513
Cl^−^	110	112	111	111	0.758	0.212
Anion Gap^1^	14.6	16.2	15.2	15.1	0.814	0.450
Apparent digestibility, %						
DM	58.0^b^	63.3^a^	62.4^a^	64.1^a^	1.856	0.014
OM	59.0^b^	64.7^a^	64.1^a^	65.8^a^	1.883	0.007
N	45.4	53.1	53.9	52.0	3.790	0.092
NDF	50.6^b^	59.6^a^	60.2^a^	62.7^a^	2.470	<0.001
ADF	37.3^b^	48.7^a^	49.6^a^	52.8^a^	3.190	<0.001
Fecal N excretion, g/d	3.33	3.18	2.81	3.04	0.418	0.579
Urinary excretion						
Total volume, L/d	2.05	2.49	1.62	1.79	0.271	0.133
N, g/d	9.15	8.65	9.92	8.13	3.840	0.605
Creatinine, μmol/kg BW^0.75^	195^a^	133^ab^	102^b^	107^b^	23.70	0.034
PD, mmol/d	13.8^a^	7.27^b^	6.80^b^	5.90^b^	1.758	<0.001
PD/Creatinine ratio	0.95^a^	0.94^a^	0.86^ab^	0.73^b^	0.085	0.043
EMPS, mmol PD/kg DOMI	43.1^a^	23.8^b^	24.5^b^	19.0^b^	4.48	<0.001
*In vitro* fermentation						
Gas production, mmol/d	40.3^b^	52.6^a^	51.6^a^	50.3^a^	4.02	0.002
CH_4_, mmol/d	0.22^b^	0.31^a^	0.27^a^	0.28^a^	0.03	0.006
CH_4_, mol/mol FOM	6.69	9.17	6.71	7.31	1.257	0.106

BW, body weight; PD, purine derivatives; EMPS, efficiency of microbial protein synthesis; DOMI, digestible OM intake.

1 During the first 10 weeks of age, goats (*N* = 36) received a daily and oral inoculation with autoclaved rumen fluid (AUT), rumen fluid from adult animals fed forage (RFF), or fed concentrate diets (RFC) or without inoculation (CTL). Means within a raw with different superscript differ (*p* < 0.05).

Similar urinary and fecal N excretions were observed across treatments (Table 2), whereas CTL animals had higher creatinine excretion than RFF and RFC animals (*p* = 0.034). The inoculation with fresh rumen fluid promoted a negative effect on the microbial protein synthesis as noted by the lower PD excretion (*p* < 0.001) and PD / creatinine ratio (*p* = 0.014) than observed for CTL animals. The efficiency of microbial protein synthesis (EMPS), measured as PD excretion divided by the digestible OM intake, was also lower for inoculated than for the CTL animals (*p* < 0.001).

### Rumen multi-kingdom microbiota

**Figure 2:**
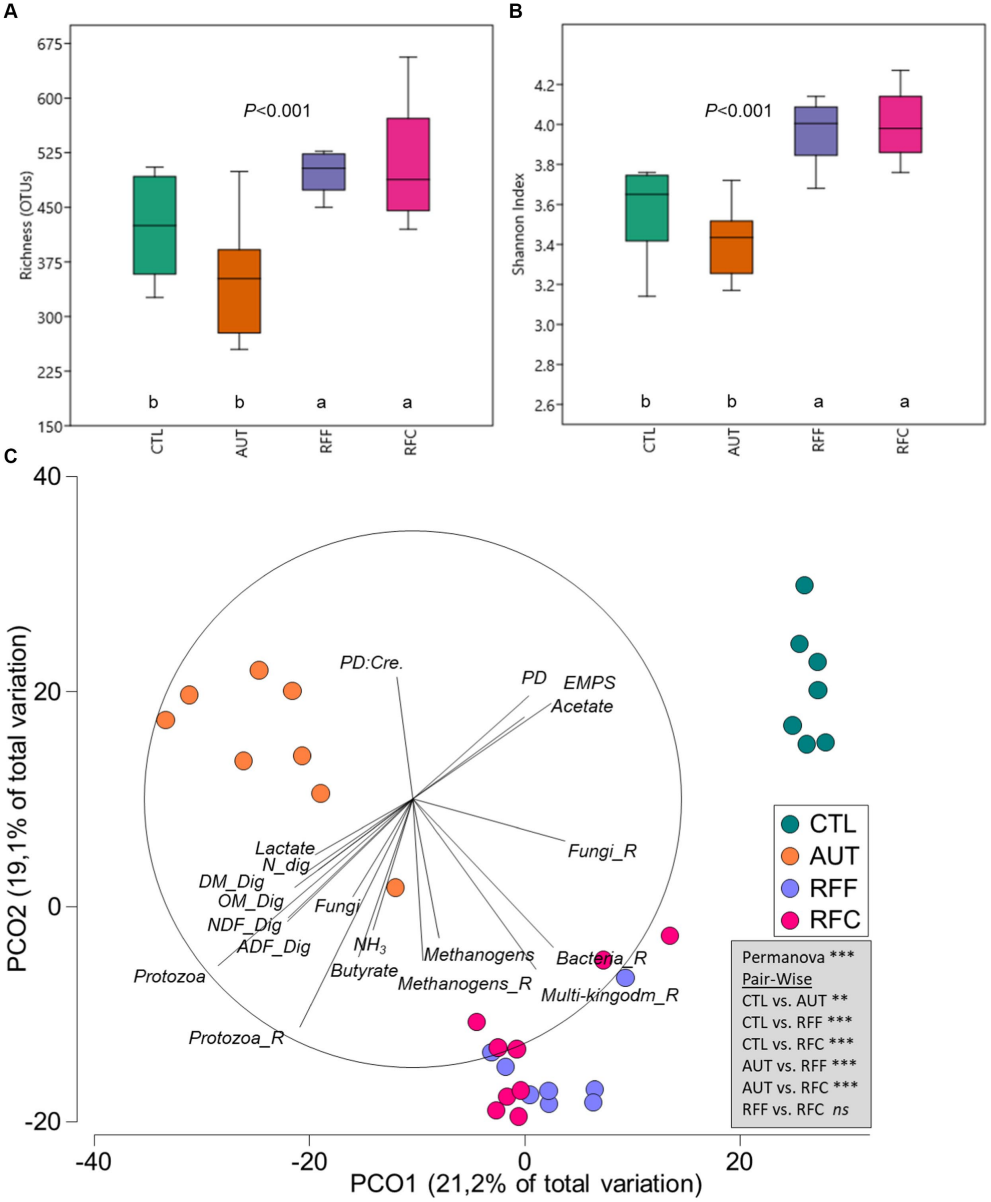
Box plot illustrating the rumen multi-kingdom diversity in terms of richness **(A)** and Shannon’s index **(B)** in 26-week-old goats measured at 21 days after a shift from a high-concentrate to a full forage diet (oats hay). During the first 10 weeks of age, goats (*N* = 36) received a daily and oral inoculation with autoclaved rumen fluid (AUT), rumen fluid from adult animals fed forage (RFF), or fed concentrate diets (RFC) or without inoculation (CTL). Principal coordinates analysis **(C)** illustrating relationships (*ρ* > 0.4) between the structure of the rumen microbiota and productive data. PERMANOVA values are provided based on the Bray–Curtis dissimilarity.

The multi-kingdom microbiota included all microbial OTUs from bacteria (87.4%), methanogens (2.7%), protozoa (2.1%) and anaerobic fungi (7.8%). Inoculation with fresh rumen fluid led to a substantial increase in the rumen microbial diversity (Figures 2A,B) in terms of richness (*p* < 0.001) and Shannon’s index (*p* < 0.001) than CTL and AUT animals. Principal coordinate analysis showed clear differences in the structure of the rumen microbiota according to the treatments (Figure 2C, *p* < 0.001). Pair-wise comparisons identified significant differences across all treatments except for RFF and RFC which showed a similar multi-kingdom microbial community structure. Tripod vectors showed that the structure of the rumen microbiota in RFF and RFC animals positively correlated with higher diversity values (for bacteria, methanogens, protozoa, anaerobic fungi and multi-kingdom) and negatively with PD to creatinine ratio. On the contrary, the multi-kingdom community structure in CTL animals positively correlated with PD excretion, EMPS and acetate molar proportion and negatively with feed digestibility and rumen protozoa, fungi, ammonia, butyrate and lactate concentrations. For a more comprehensive description of the rumen microbiota, the main microbial groups were studied separately.

### Rumen bacteria

**Figure 3:**
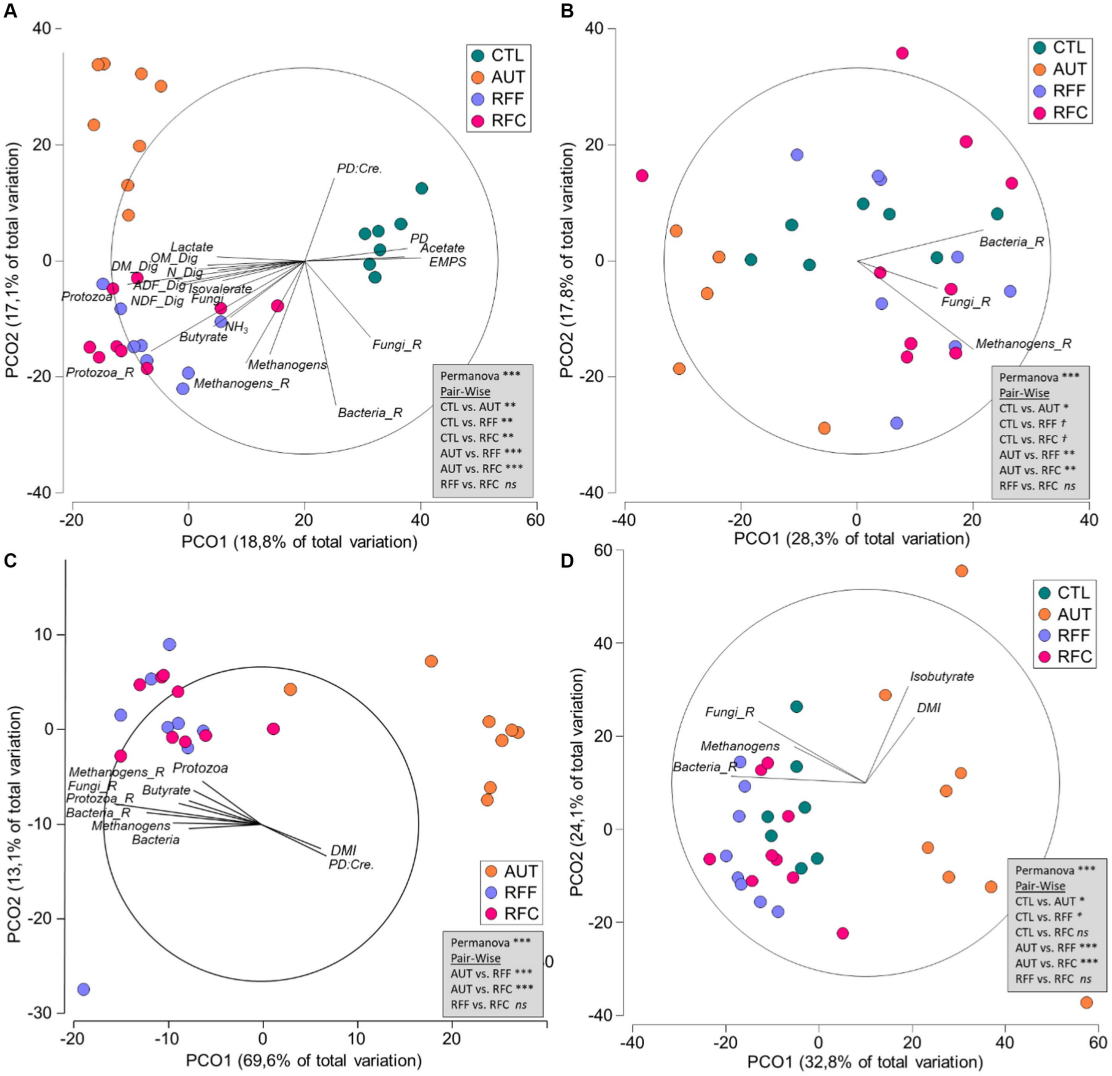
Principal Coordinate Analysis illustrating the structure of the rumen bacteria **(A)**, methanogens **(B)**, protozoa **(C)** and anaerobic fungi community **(D)** in 26-week-old goats measured at 21 days after a shift from a high-concentrate to a full forage diet (oats hay). During the first 10 weeks of age, goats (*N* = 36) received a daily and oral inoculation with autoclaved rumen fluid (AUT), rumen fluid from adult animals fed forage (RFF), or fed concentrate diets (RFC) or without inoculation (CTL). Relationships (*ρ* > 0.4) between the structure of the rumen microbiota and productive data are shown along with PERMANOVA values based on the Bray–Curtis dissimilarity.

Quantitative PCR showed that the bacterial community represented the most abundant microbial group in the rumen and its concentration was similar across treatments (Table 3). Bacterial sequencing yielded 9,758 ± 2,657 high-quality sequences per sample and diversity analysis showed that treatments RFF and RFC had the highest bacterial richness (*p* < 0.001), Shannon’s (*p* < 0.001) and Simpson’s indexes (*p* = 0.061). The AUT animals had the lowest bacterial richness across treatments but similar Shannon and Simpson index than CTL animals. The bacterial community structure (Figure 3A) showed a clear separation between inoculated (RFF, RFC and AUT, left) and non-inoculated animals (right) according to the PCO1. PERMANOVA analysis showed that the structure of the rumen bacterial community was significantly affected by the treatments (*p* < 0.001) and pair-wise comparisons detected significant differences across all treatments except for RFF and RFC animals. Moreover, tripod vectors showed that the bacterial community structure was intimately correlated with rumen fermentation and productive variables. In particular, the bacterial community in RFF and RFC animals positively correlated with higher feed digestibility (DM, OM, NDF and ADF), rumen ammonia, lactate, butyrate, methanogens, protozoa and anaerobic fungal levels as well as protozoa and methanogens diversities, whereas in CTL animals it was positively correlated with higher acetate molar proportion and microbial protein synthesis (in terms of PD, PD to creatinine ratio and EMPS). Bacterial community structure in AUT animals was negatively correlated with the bacterial and fungal diversities.

**Table 3:** Rumen bacterial diversity and taxa abundances in 26-week-old goats measured at 21 days after a shift from a high-concentrate to a full forage diet (oats hay).

Treatments^1^	CTL	AUT	RFF	RFC	SED	*p*-value
Concentration, log10 copy/mg DM	10.5	10.3	10.0	10.4	0.263	0.367
Diversity						
Richness, OTUs	362^b^	283^c^	409^a^	415^a^	29.80	<0.001
Shannon	3.63^b^	3.49^b^	4.22^a^	4.26^a^	0.199	<0.001
Simpson	0.91^b^	0.91^b^	0.95^a^	0.96^a^	0.023	0.061
Abundance, %						
Ratio Firmicutes/Bacteroidota	0.67^a^	0.28^b^	0.70^a^	0.53^a^	0.105	0.003
*p_Actinobacteriota*	0.01^b^	0.00^b^	0.00^b^	0.05^a^	0.019	0.003
*p_Bacteroidota*	57.8^b^	72.2^a^	50.7^b^	57.2^b^	4.226	<0.001
*f_Bacteroidales_RF16_group*	3.56^bc^	10.2^a^	1.81^c^	5.35^b^	1.682	<0.001
*f_Bacteroidetes_BD2-2*	0.12^a^	0.01^b^	0.18^a^	0.13^a^	0.047	0.021
*f_p-251-o5*	0.00^b^	0.01^b^	0.30^ab^	0.68^a^	0.255	0.001
*f_p-2534-18B5_gut_group*	4.38^a^	0.01^b^	4.27^a^	0.64^ab^	1.709	0.008
*f_Prevotellaceae*	34.1^b^	50.0^a^	31.3^b^	35.4^b^	4.495	0.006
*f_Rikenellaceae*	5.87^ab^	2.95^b^	6.05^ab^	8.96^a^	1.915	0.031
*p_Chloroflexi*	0.00^b^	0.41^a^	0.02^b^	0.01^b^	0.140	<0.001
*p_Cyanobacteria*	1.79^b^	0.93^b^	2.85^ab^	5.20^a^	1.371	0.035
*p_Desulfobacterota*	0.04^a^	0.00^b^	0.00^b^	0.00^b^	0.008	<0.001
*p_Elusimicrobiota*	0.09^ab^	0.02^b^	0.37^ab^	0.39^a^	0.148	0.010
*p_Fibrobacterota*	1.38^b^	0.48^b^	6.03^a^	2.40^b^	1.249	<0.001
*p_Firmicutes*	36.2^a^	19.8^b^	33.2^b^	29.4^b^	3.653	0.005
*f_Acholeplasmataceae*	0.00^b^	0.01^b^	0.30^a^	0.08^ab^	0.094	0.005
*f_Christensenellaceae*	5.52^a^	1.45^b^	0.77^b^	0.83^b^	1.004	0.002
*f_Clostridia_UCG-014*	1.62^a^	0.24^b^	0.74^b^	0.92^ab^	0.327	0.006
*f_Clostridia_vadinBB60_group*	0.27^bc^	0.16^c^	0.62^a^	0.54^ab^	0.134	0.031
*f_Erysipelatoclostridiaceae*	0.17^b^	0.19^b^	12.1^a^	8.90^a^	3.353	<0.001
*f_Izemoplasmatales*	0.02^ab^	0.00^b^	0.03^a^	0.00^b^	0.012	0.033
*f_Oscillospiraceae*	16.7^a^	0.50^b^	0.99^b^	3.61^b^	3.626	0.001
*f_Selenomonadaceae*	1.16^b^	10.2^a^	5.13^b^	4.40^b^	1.846	<0.001
*f_UCG-010*	0.04^b^	0.06^b^	0.33^a^	0.16^b^	0.061	0.002
*p_Proteobacteria*	1.50	1.71	2.52	1.87	0.853	0.194
*f_Paracaedibacteraceae*	0.00^b^	0.00^b^	0.08^a^	0.08^a^	0.042	0.011
*p_Spirochaetota*	0.91	0.45	1.65	1.12	0.383	0.066
*p_Synergistota*	0.14^b^	3.65^a^	2.35^ab^	2.06^ab^	0.977	0.023
*p_Verrucomicrobiota*	0.01^b^	0.28^a^	0.27^a^	0.22^a^	0.059	0.002

1 During the first 10 weeks of age, goats (*N* = 36) received a daily and oral inoculation with autoclaved rumen fluid (AUT), rumen fluid from adult animals fed forage (RFF), or fed concentrate diets (RFC) or without inoculation (CTL). Means within a raw with different superscript differ (*p* < 0.05). Only families (f) with an average abundance higher than 0.01% and *p*-values <0.1 are shown.

The analysis of the relative abundances of the most predominant bacterial taxa showed a strong effect of the treatments (Table 3). Control animals showed the highest abundances for the phyla Actinobacteriota, Desulfobacterota and Firmicutes (including the families *Christensenellaceae, Clostridia_UCG-014* and *Oscillospiraceae*), whereas AUT animals had the highest abundances for the phyla Bacteroidota (including the family Prevotellaceae) and Synergistota and the family Selenomonadaceae, resulting on the lowest Firmicutes / Bacteroidota ratio across treatments. Inoculation with RFF in early life led to the highest abundances for Fibrobacterota, Acholeplasmataceae, Izemoplasmatales and UCG-010, whereas inoculation with RFC did it for Actinobacteriota, p-251-o5, Rikenellaceae, Cyanobacteria and Elusimicrobiota. Moreover, all animals inoculated with fresh rumen fluid (RFF or RFC) had the highest abundances for Bacteroidetes_RF16_group, Erysipelatoclostridiaceae and Paracaedibacteraceae.

### Rumen methanogens

No differences were noted in the rumen methanogens concentrations across treatments (Table 4). Methanogens sequencing yielded 7,147 ± 702 high quality sequences per sample and treatment AUT led to the lowest methanogens richness (*p* = 0.008), Shannon’s (*p* = 0.005) and Simpson’s indexes (*p* = 0.009) across treatments. Control animals also had lower methanogens richness than that observed for RFF and RFC animals, but similar for Shannon’s and Simpson’s indexes, suggesting the presence of fewer species but with a higher homogeneity in their abundance. The methanogens community structure was affected by the treatments (Figure 3B, *p* < 0.001). Pair-wise comparisons showed that AUT animals had a different methanogens community than the rest of the animals (*p* < 0.05) whereas RFF and RFC tended to differ to the CTL animals (*p* < 0.1). A similar methanogens community structure was observed for RFF and RFC animals and it was positively correlated with the bacteria, methanogens and anaerobic fungal richness. Interestingly, the structure of the methanogens community did not correlate with the metadata considered in this study.

**Table 4:** Rumen methanogens diversity and taxa abundances in 26-week-old goats measured at 21 days after a shift from a high-concentrate to a full forage diet (oats hay).

Treatments^1^	CTL	AUT	RFF	RFC	SED	*p*-value
Concentration, log10 copy/mg DM	6.60	6.85	6.83	7.24	0.343	0.218
Diversity						
Richness, OTUs	8.67^b^	9.00^b^	13.5^a^	11.0^ab^	1.452	0.008
Shannon	1.32^a^	0.92^b^	1.37^a^	1.32^a^	0.128	0.005
Simpson	0.66^a^	0.47^b^	0.62^a^	0.64^a^	0.056	0.009
Abundance^1^, %						
f_Methanobacteriaceae	17.4	22.2	26.4	23.6	3.768	0.764
f_Methanocaldococcaceae	0.00	0.00	0.15	0.28	0.091	0.187
*g_Methanocaldococcus*	0.00	0.00	0.00	0.28	0.077	0.058
f_Methanomicrobiaceae	3.73	0.00	0.00	3.57	1.657	0.534
f_Methanomassiliicoccaceae	78.9	77.8	73.2	72.6	3.639	0.838
*s_Group8_sp WGK1*	0.00	0.05	1.81	0.71	0.521	0.088
*s_Group10_sp*	18.5	3.99	4.63	6.34	2.446	0.053
*s_Group11_sp ISO4-G11*	19.1^a^	0.00^b^	18.8^a^	18.5^a^	4.697	0.018
*g_Group9*	34.5^b^	71.4^a^	43.8^ab^	40.4^ab^	6.052	0.041
*s_Group9_sp CH1270*	0.00^b^	0.00^b^	0.01^b^	0.43^a^	0.125	0.046
*s_Group9_sp ISO4-G1*	34.3^b^	71.4^a^	43.8^ab^	40.0^ab^	6.049	0.035

1 During the first 10 weeks of age, goats (*N* = 36) received a daily and oral inoculation with autoclaved rumen fluid (AUT), rumen fluid from adult animals fed forage (RFF), or fed concentrate diets (RFC) or without inoculation (CTL). Means within a raw with different superscript differ (*p* < 0.05).

The abundances of the main methanogens families were unaffected by the treatments (Table 4), but moderate differences were noted within the Methanomassilicoccaceae family. The methanogens species *Group10_sp* and *Group9_sp ISO4-G1* showed the highest abundances in the CTL and AUT animals, respectively. Inoculation with RFF and with RFC increased the abundance of *Group8_sp WGK1* and *Group9_sp CH1210*, respectively, whereas this later group was undetected in the rest of the treatments.

### Rumen protozoa

Control animals remained protozoa-free during the entire duration of this experiment, while no differences in the rumen protozoal concentration were detected across the other three treatments (Table 5). Protozoal sequencing yielded 20,121 ± 4,504 high quality sequences per sample and a substantially lower protozoal diversity indexes were noted for AUT than for RFF or RFC animals (*p* < 0.001). Similarly, PCoA and PERMANOVA analyses also showed a different protozoal community structure for AUT than for RFF or RFC animals (*p* < 0.001, Figure 3C). The protozoal community structure in RFF and RFC was similar (*p* > 0.1) and positively correlated with the rumen concentration of bacteria, methanogens and protozoa, the butyrate molar proportion and the bacteria, methanogens, protozoa and anaerobic fungal diversity. On the contrary, the protozoal structure in AUT animals positively correlated with DMI and PD to creatinine ratio. The protozoal community in the AUT animals was more abundant in the subfamily Entodiniinae (*p* < 0.001, average 96.2%) whereas animals inoculated with fresh rumen fluid had higher abundances of *Enoploplastron_triloricatum, Ophryoscolex_sp_LDK-2011,* and the family Isotrichidae, including *Dasytricha, Isotricha prostoma* and *Isotricha intestinalis*.

**Table 5:** Rumen protozoal diversity and taxa abundances in 26-week-old goats measured at 21 days after a shift from a high-concentrate to a full forage diet (oats hay).

Treatments^1^	CTL	AUT	RFF	RFC	SED	*p*-value
Concentration, log10 copy/mg DM	ND	8.79	8.38	9.02	0.430	0.348
Diversity						
Richness, OTUs	ND	20.5^b^	31.3^a^	32.9^a^	1.085	<0.001
Shannon	ND	1.68^b^	2.24^a^	2.28^a^	0.062	<0.001
Simpson	ND	0.76^b^	0.86^a^	0.86^a^	0.014	<0.001
Abundance, %						
f_Ophyoscolecidae	ND	97.5^a^	77.5^b^	81.6^b^	3.087	<0.001
sf_Entodiniinae; *g_Entodinium*	ND	96.2^a^	74.9^b^	79.1^b^	3.312	<0.001
sf_Diplodiniinae	ND	1.33	2.34	2.36	0.423	0.138
*g_Enoploplastron_triloricatum*	ND	0.00^b^	0.49^a^	0.32^a^	0.114	0.003
*g_Polyplastron*	ND	1.32	1.73	1.57	0.397	0.600
*g_Diplodinium*	ND	0.01	0.12	0.47	0.171	0.073
sf_Ophryoscolecinae	ND	0.00^b^	0.23^a^	0.14^a^	0.053	0.003
*s_Ophryoscolex_sp_LDK-2011*	ND	0.00^b^	0.22^a^	0.14^a^	0.052	0.003
f_Isotrichidae	ND	1.14^b^	21.85^a^	16.6^a^	3.099	<0.001
*g_Dasytricha*	ND	0.92^b^	12.15^a^	12.8^ab^	2.063	0.001
*g_Isotricha*	ND	0.23^b^	9.70^a^	3.84^a^	2.372	<0.001
*s_Isotricha_prostoma*	ND	0.15^b^	6.00^a^	1.66^a^	1.484	<0.001
*s_Isotricha_intestinalis*	ND	0.06^b^	2.63^a^	1.15^a^	0.585	<0.001

1 During the first 10 weeks of age, goats (*N* = 36) received a daily and oral inoculation with autoclaved rumen fluid (AUT), rumen fluid from adult animals fed forage (RFF), or fed concentrate diets (RFC) or without inoculation (CTL). Means within a raw with different superscript differ (*p* < 0.05).

### Anaerobic fungi

Anaerobic fungal concentration in the rumen was similar across treatments (Table 6). Fungal sequencing yielded 21,298 ± 6,920 high quality sequences per sample, but only the anaerobic fungal sequences (phylum Neocallimastigomycota) were further considered (19,267 ± 5,647 per sample). Animals inoculated with AUT showed the lowest anaerobic fungal diversity in terms of richness, Shannon’s and Simpson’s indexes (Table 6) across treatments. Treatment AUT promoted a particular rumen fungal community structure (Figure 3D) which was positively correlated with the DMI and rumen isobutyrate molar proportion. On the contrary, similar fungal community structure was observed for the treatments RFF, RFC and CTL having a positive correlation with the bacterial and fungal richness and the rumen methanogens concentration. Over half of the anaerobic fungal sequences were unclassified at the genus level. Treatment AUT tended to increase the rumen abundances of *Caecomyces* and *Anaeromyces* while led to the lowest levels of *Neocallimastix*. On the contrary, treatment RFC led to the highest *Neocallimastix* abundance, the lowest *Caecomyces* abundance and the absence of *Anaeromyces*. Moreover, CTL animals also had lower *Neocallimastix* abundance than RFC animals.

**Table 6:** Rumen anaerobic fungal diversity and taxa abundances in 26-week-old goats measured at 21 days after a shift from a high-concentrate to a full forage diet (oats hay).

Treatments^1^	CTL	AUT	RFF	RFC	SED	*p*-value
Concentration, log10 copy/mg DM	6.62	7.20	7.18	7.33	0.352	0.234
Diversity						
Richness, OTUs	52.5^a^	36.6^c^	43.9^bc^	49.2^ab^	3.910	0.001
Shannon	2.47^a^	2.02^b^	2.48^a^	2.55^a^	0.143	0.002
Simpson	0.84^a^	0.77^b^	0.87^a^	0.86^a^	0.036	0.041
Abundance, %						
g_Anaeromyces	0.77	0.91	0.75	0.00	0.292	0.096
g_Caecomyces	25.3^ab^	30.9^a^	20.8^ab^	6.82^b^	4.903	0.047
g_Neocallimastix	16.6^b^	6.23^c^	25.2^ab^	33.6^a^	3.207	<0.001
Unclassified	57.4	61.9	53.3	59.6	3.763	0.820

1 During the first 10 weeks of age, goats (*N* = 36) received a daily and oral inoculation with autoclaved rumen fluid (AUT), rumen fluid from adult animals fed forage (RFF), or fed concentrate diets (RFC) or without inoculation (CTL). Means within a raw with different superscript differ (*p* < 0.05).

### Correlation analysis

Total VFA concentration was not associated with relevant microbiological changes (Table 7), however the acetate molar proportion was positively correlated with seven (including *Coprococcus, Desulfobacteria* and *CAG-352*) and negatively correlated with six bacterial taxa (e.g., *Lachnospiraceae_ND3007_group and Prevotellaceae_UCG-001*) and two protozoal taxa (e.g., *Ophryoscolex*) as well as with the protozoal richness. Propionate molar proportion only correlated positively and negatively with two and three bacterial species, respectively. On the contrary butyrate molar proportion was highly associated with changes in the rumen microbiota and had positive correlations with several protozoal features (including protozoal concentration, richness and abundances of Isotricha, Dasytricha, Enoploplastron and Ophryoscolex), methanogens richness, multi-kingdom richness and eight bacterial taxa (including *Lachnospiraceae_ND3007_group*), whereas negative correlation were detected with seven bacterial and one fungal taxa. Although rumen ammonia concentration and butyrate molar proportion had a small correlation coefficient (*ρ* = 0.32, *p* = 0.072), 15 out of the 19 microbial variables that correlated with the ammonia were the same (and in the same sign) than those that correlated with butyrate (including 11 protozoal variables). Rumen lactate concentration was negatively correlated with the abundance of 10 bacterial taxa but positively correlated with *Dasytricha* and *Entodinium* abundances. Urinary PD excretion and the EMPS were negatively correlated with *Entodinium* and several bacterial taxa (*Oligosphaeraceae, Anaeroplasma* and *Lachnospiraceae_ND3007_group, Paracaedibacteraceae* and *Selenomonas*) but also had a positive correlation with other bacterial taxa (including, *Oscillospira*, *Prevotella ruminicola* and *Oscillospira_guilliermondii*). The N digestibility correlated with the same bacterial taxa than described for the rumen NH_3_ concentration, however this was not the case for the protozoal taxa since the abundances of *Diplodinium* and *Entodinium* positively correlated to N digestibility but not with NH_3_ concentration. Finally, DM digestibility and NDF digestibility mostly correlated with the same rumen microbes given the high proportion of fiber in the diet. Several bacterial (including *Prevotellaceae_UCG-003*, *Lachnospiraceae_ND3007_group*, *Quinella*, *Fretibacterium*), methanogens (*Group9_spCH1270*) and protozoal taxa (including *Diplodinium* and *Entodinium*) as well as the protozoal concentration and richness had a positive correlation with NDF digestibility, whereas up to 11 bacterial taxa showed a negative correlation, being most of them amylolytic species.

**Table 7:** Spearman’s correlations (*ρ* > 0.4, *p* < 0.001) between the rumen microbes and productive data.

Kingdom	Taxa	Ace.	Prop.	But.	VFA	NH_3_	Lact	PD	DMd	Nd	NDFd	EMPS
Multiple	Multi-kingdom richness			0.49								
Bacteria	*p_Desulfobacterota*	0.52		−0.44		−0.40					−0.45	0.42
Bacteria	*p_Verrucomicrobiota*					0.49				0.53		
Bacteria	*f_Christensenellaceae*			−0.58		−0.48						
Bacteria	*f_Erysipelatoclostridiaceae*			0.51		0.49						
Bacteria	*f_Oligosphaeraceae*							−0.53				−0.56
Bacteria	*f_Oscillospiraceae*	0.41					−0.52				−0.40	
Bacteria	*f_p-251-o5*			0.45			0.47					
Bacteria	*f_Paracaedibacteraceae*			0.59								−0.42
Bacteria	*f_RF39*						−0.54		−0.62		−0.63	
Bacteria	*g_Acetitomaculum*		−0.42		−0.40		−0.43					
Bacteria	*g_Anaeroplasma*	−0.45		0.50	0.44			−0.40				
Bacteria	*g_CAG-352*	0.59	−0.41	−0.40			−0.58	0.43		−0.40	−0.55	0.44
Bacteria	*g_Coprococcus*	0.63	−0.5	−0.45				0.44			−0.54	0.45
Bacteria	*g_Fretibacterium*	−0.48		0.40							0.40	
Bacteria	*g_Lachnospiraceae_ND3007*	−0.53		0.63				−0.45			0.41	−0.40
Bacteria	*g_Oscillospira*	0.47		−0.35				0.55				0.56
Bacteria	*g_p-2534-18B5_gut_group*						−0.36				−0.42	
Bacteria	*g_Prevotellaceae_Ga6A1_group*						−0.48	0.42			−0.54	0.44
Bacteria	*g_Prevotellaceae_UCG-001*	−0.56	0.45									
Bacteria	*g_Prevotellaceae_UCG-003*								0.42		0.44	
Bacteria	*g_Quinella*	−0.48	0.53						0.40		0.43	
Bacteria	*g_RF39*						−0.54		−0.62		−0.63	
Bacteria	*g_Selenomonas*					0.47						−0.40
Bacteria	*g_Succinivibrionaceae_UCG-002*	0.40					−0.43					
Bacteria	*g_UCG-002*						−0.59				−0.52	0.44
Bacteria	*g_vadinBE97*					0.73						
Bacteria	*g_Veillonellaceae_UCG-001*	−0.41		0.53		0.59						
Bacteria	*s_bacterium_FB2012*						−0.44		−0.43	−0.48	−0.53	
Bacteria	*s_Fibrobacter_succinogenes*			0.59						0.43		
Bacteria	*s_Oscillospira_guilliermondii*	0.42		−0.46				0.47				0.46
Bacteria	*s_Prevotella_ruminicola*							0.53			−0.41	0.44
Methanogen	Methanogens richness			0.43						0.44		
Methanogen	*Group9_spCH1270*								0.41		0.43	
Protozoa	Protozoal concentration			0.66		0.49					0.52	
Protozoa	Protozoal richness	−0.46		0.62		0.66					0.44	
Protozoa	*g_Dasytricha*			0.67		0.49						
Protozoa	*g_Diplodinium*						0.55		0.5	0.46	0.49	
Protozoa	*g_Enoploplastron*			0.55		0.49						
Protozoa	*g_Entodinium*							−0.51	0.45	0.40	0.48	−0.54
Protozoa	*g_Isotricha*			0.61		0.58						
Protozoa	*g_Ophryoscolex*	−0.50		0.41		0.54						
Protozoa	*g_Polyplastron*	−0.41				0.51			0.45	0.48		
Protozoa	*s_Enoploplastron_triloricatum*			0.55		0.49						
Protozoa	*s_Entodinium_sp_LDK-2011*					0.53	0.44		0.47		0.46	−0.40
Protozoa	*s_Isotricha_intestinalis*	−0.45		0.66		0.55						
Protozoa	*s_Isotricha_prostoma*			0.56		0.58						
Fungi	*g_Caecomyces*			−0.50								

Ace, Acetate; Pro, Propionate; But, Butyrate; Lact, Lactate; PD, Purine derivatives; d, digestibility; EMPS, Efficiency of microbial protein synthesis. Data across all experimental treatments measured in 26-week-old (*N* = 36) measured at 21 days after a shift from a high-concentrate to a full forage diet (oats hay).

## Discussion

This study demonstrated that the inoculation of young ruminants during the pre-weaning period with rumen fluid from adult ruminants has long-term effects on the rumen microbiota and certain physiological implications in terms of feed efficiency and health when animals are fed forage.

### Effects of different microbial inocula

Adult animals generally show a high host specificity which makes difficult to permanently modify their rumen microbiota (33). As a result, Weimer et al. (34) demonstrated that after a near-total exchange of rumen content among adult cows, they were able to re-establish their initial rumen bacterial community and fermentation pattern after 14–61 days. On the contrary, it has been suggested that nutritional interventions in early life can represent an opportunity to modulate the rumen microbial colonization having short and potentially long-term effects on the rumen microbial community structure and animal productivity (33). A companion publication of the present experiment (13) and similar studies described the positive short-term effects of inoculating fresh (11, 12) or lyophilized (14, 15) rumen fluid from adult ruminants to young ruminants. In the present study we demonstrated that these effects have a prolonged persistency since after a substantial time elapsed (28 weeks of age) the RFF and RFC animals still had a higher rumen multi-kingdom microbial community richness (503 OTUs) as a result of a higher bacterial and protozoal diversity than the CTL animals (423 OTUs) which remained protozoa-free. An incomplete rumen microbial colonization has been described in artificially-reared ruminants without physical contact with adult animals (8), and characterized by absence of rumen protozoa, as they are highly sensitive to oxygen, making it necessary to have direct contact between animals for an effective transmission (35). The AUT animals also retained low bacterial, anaerobic fungi and protozoal richness, which was dominated by a single protozoa genus (*Entodinium*, 96.2%). This observation indicated an incomplete protozoal colonization, possibly as a result of a cross-faunation that may have accidentally occurred when inoculating animals from different groups (13). However, the presence of few protozoal species in AUT animals, along with the potential positive effects derived from the inoculation with rumen metabolites such as VFA (36), micro-nutrients and microbial extracts (37) could explain the moderate positive impact on the energy metabolism (feed digestibility and gas production) along with the negative effect on the protein metabolism (lower microbial protein synthesis than CTL goats).

In relation with the type of microbial inocula, De Barbieri et al. (38) showed a different rumen bacterial colonization when young lambs were inoculated with rumen fluid from adult sheep supplemented with coconut oil or protected fat. Similarly, our companion paper showed that inoculation with RFF and RFC also led to certain differences in the rumen microbial community up to weaning (13). Thus, it was hypothesized that inoculation with a more diverse bacterial inocula adapted to the forage digestion (RFF) could be beneficial for an efficient forage utilization in adult life. Unfortunately, our results suggest that animals inoculated with RFF or RFC had similar rumen microbiota. This microbial convergence could be a partially explained by an adaptation to the diet given the high plasticity of the rumen microbiota (39). This observation suggests that the long-germ effects of using inocula with different microbial composition are negligible since only those microbes able to survive in the rumen environment will ultimately flourish independently of their abundance in the initial inocula (40).

### Rumen microbial fermentation

Fiber fermentation is a complex process which requires the combined action of multiple microbial groups (41), therefore it may be expected that a greater rumen microbial diversity would favor the adaptation and utilization of fibrous diets by the host. Our study showed that all animals were ultimately adapted to the forage diet without experiencing stress or digestive disorders based on the similar levels of hair cortisol, blood metabolites and absence of diarrhea across treatments (42). However, this adaptation process was substantially faster for RFF and RFC given their higher DMI and rumen VFA concentration than reported for CTL animals at 4 d after the dietary shift, possibly as a result of a higher forage degradation by the rumen microbes (43).

The presence of a complex rumen microbial community in RFF and RFC animals also promoted higher butyrate (+36%) and propionate (+10%) molar proportions in detriment to acetate (−6.1%) after the 21 days of adaptation to the diet, mostly as a result of a complex rumen protozoal community (44). The ability of protozoa to engulf carbohydrates and exogenous fatty acids may divert more carbon toward VFA production, butyrate being the main fermentation product derived from the protozoal activity (45). This observation was confirmed by positive correlation observed between the butyrate proportion and several protozoal variables including concentration, richness and abundance of holotrich protozoa (*Isotricha* and *Dasytricha*). Holotrich protozoa, which represented 20% of the protozoa in RFF and RFC animals, have a limited ability to digest fiber (46) and to predate bacteria in the rumen (47). However, they exhibit a chemotaxis to simple sugars (48) which are fermented into butyrate, CO_2_ and H_2_ as the main fermentation products. This H_2_ production favors the inter-species H_2_ transfer toward protozoal epi- and endo-symbiotic methanogens (49, 50), and it could explain the high butyrate and CH_4_ production observed in holotrich-monofaunated sheep (51) as well as in the RFF and RFC animals (+26%) in comparison to the CTL animals.

Over the last decade, important research efforts have been focussed on studying rumen microbiota and their correlations with the feed efficiency and the overall biology of the host. After analyzing the rumen microbiota of 146 dairy cows fed concentrate feeds, Shabat et al. (52) concluded that cows that feed efficient cows (in terms of feed conversion ratio) had higher propionate, butyrate, valerate and isovalerate molar proportions, increased rumen abundances of *Megasphaera elsdenii* and *Coprococcus catus*, as well as lower bacterial diversity and CH_4_ emissions than less efficient cows. On the contrary, Myer et al. (53) reported no differences in rumen bacterial diversity between steers differing in feed conversion ratio. Likewise, Lopes et al. (54) observed similar rumen bacterial and fungal diversities, but higher archaeal diversity and Bacteroidetes to Firmicutes ratio in feed efficient Nellore steers. The discrepancy between studies seems to rely on type of diets consumed by the ruminants indicating that it is unlikely to find a one-size-fits-all approach to optimize the rumen function across the different ruminant production systems. Our correlation analysis based on animals fed forage identified two distinctive types of rumen microbes according to their activity: (i) butyrate producers that positively correlated with butyrate and negatively with acetate molar proportions such as, *Ophryoscolex*, *Isotricha intestinalis*, *Anaeroplasma*, *Lachnospiraceae_ND3007_group*, *Veillonellaceae_UCG-001*, *Fretibacterium* and protozoal richness, and (ii) acetate producers that positively correlated with acetate and negatively with butyrate including *Desulfobacterota*, *Coprococcus, Oscillospira* and *CAG-352*. On the contrary, the increased propionate molar proportion observed in RFF and RFC animals (+10%) was not associated with rumen microbiological changes suggesting that it may be a beneficial but indirect effect derived from compositional changes in other VFA proportions. This indirect effect was exemplified with *Coprococcus*, that is one of the few rumen bacteria able to degrade phloroglucinol into acetate as unique fermentation product (55), and in our study it had a strong positive correlation with acetate but also an indirect negative correlations with propionate and butyrate molar proportions. Rumen lactate concentration was unaffected by the treatments, however up to 10 bacterial taxa had strong negatively correlations with lactate concentration, including the starch utilizer *Succinivibrionaceae_UCG-002* and Oscillospiraceae. This observation indicates that lactate may have been transformed into propionate in RFF and RFC as a result of a higher fiber digestion and availability of simple sugars (56). Similarly, the increased valerate molar proportion in RFF and RFC animals (+20%) could be linked to a higher digestion of glucose, starch and cellulose (57). Increased Firmicutes to Bacteroidota ratio, as noted in RFF and RFC animals, has been correlated to higher DMI, BW gain and feed efficiency in beef cattle (58), but not in dairy cows (54) possibly as a result of differences in the type of diets. These findings suggest that inoculation with fresh rumen fluid led to a shift toward an energetically efficient carbohydrate fermentation. This shift is attributed to the increased production of propionate and butyrate, which offer higher energy yields, release less H_2,_ and enhance the host’s energy utilization efficiency compared to acetate production (59). However, this nutritional approach faces significant limitations. In practice, inoculating rumen fluid is not feasible under on-farm conditions due to management and health issues such as the presence of potential pathogens; moreover, gaining access to fistulated animals poses its own set of challenges. Therefore, it becomes imperative to explore alternative nutritional and management strategies to enhance feed utilization in ruminants fed forage diets. These may include the use of probiotics, dietary diversification, or the introduction of adult companions to foster a more diverse rumen microbial community (8).

### Rumen microbiota and feed digestion

Most of forage sources used in the Mediterranean basin, as the one used in this study (oats hay) are preserved as hay due to the low precipitation and high seasonality resulting on low quality and highly fibrous forages. These peculiarities make that increasing forage digestibility represent a highly desirable attribute to increase productivity is such conditions. It has been demonstrated that forage digestion is linked with the forage microbial colonization, which is a three-steps process and of greater complexity for preserved than for fresh forages (60). The primary feed colonization is initiated by the rumen microbes associated with the liquid phase. Due to their high motility, protozoa are the microbes that most rapidly colonize forage during the primary colonization (60). Thus, the presence of a complex protozoal community in RFF and RFC animals, along with higher bacterial and methanogens diversities, could accelerate this primary colonization favoring the DM (+8.9%) and OM (+10%) digestibility. A meta-analysis reported similar increases in OM digestibility (+5%), total VFA (+5%) and CH_4_ emissions (+13%) in presence of rumen protozoa (44), as noted in our study.

Once feed is colonized by protozoa, the soluble plant components act as chemo-attractants, allowing secondary colonizers such as fungal zoospores and bacteria to further colonize feed particles (61). In this sense, inoculation with fresh rumen fluid led to lower abundance of *Anaeromyces* and *Caecomyces* which have a preference for glucose and fructose (62) but higher of *Neocallimastix* which is a monocentric fungus able to utilize a wider spectrum of substrates including cellulose, xylose, glucose, starch, grass and straw (63). The ability of anaerobic fungi to form resistant spores that allow them to retain viability in dung, soil and feed, may explain the similar anaerobic fugal concentration and diversity observed across treatments (64). Moreover this anaerobic fungal concentration was higher (2 logs) than we previously reported in ruminants fed concentrate diets suggesting an active role in fiber degradation (65). Anaerobic fungi, due to their long life cycle and they ability to digest recalcitrant lignocellulosic substrates, are efficient feed degraders (66) and able to penetrate the plant cuticle providing additional sites for bacteria to attach to protected plant tissues (60). The positive correlation observed between the fungal community structure in RFF and RFC and the bacterial richness seems to support this microbial symbiosis. This cooperation between different microbial groups could explain the higher NDF (+22%) and ADF digestibility (+37%) observed in RFF and RFC than in CTL animals. In particular, the correlation analysis identified the rumen protozoa as the key microbes for fiber digestion. Williams and Coleman (67) described high endoglucanase and xylanase activity for large *Ophryoscolecide* such as *Epidinium, Ophryoscolex*, *Enoploplastron*, *Polyplastron* and *Eudiplodinium*, weak activity for *Entodinium* and negligible fibrolytic activity for Isotrichidae. These carbohydrate-active enzymes (CAZymes) activities have recently been confirmed based on the study of the protozoal proteome and metagenome (68, 69), and here they are supported by our correlation analysis. A recent meta-analysis indicated that the effects of the absence of rumen protozoa tend to decrease as time progress after the defaunation as a result of a partial compensation by other microbial groups (70).

Methanogens are able to utilize H_2_ and prevent its accumulation during the fiber degradation process (71). Considering that between 9 and 25% of rumen methanogens are protozoal epi- and endo-symbiotic microbes (72), the presence of rumen protozoa also increased the methanogens concentration and diversity as previously shown (8, 73). This intimate association made challenging to elucidate the specific effects of each microbial group and to link changes in the methanogens community with rumen fermentation and *in vitro* CH_4_ production. Direct *in vivo* measurements of CH_4_ emissions, as well as a more detailed analysis of the H_2_ fluxes in the rumen, would be needed to better explore the impact of methanogens on the energy metabolism.

### Rumen microbiota and N metabolism

The availability of dietary protein is often a limiting factor for productivity when ruminants are fed low quality forages (74). The dietary shift from concentrate to forage feeding represented a substantial N shortage, derived from the lower CP content (from 19.9 to 7.9%) and N digestibility, which required an adaptation process. As a result, a decrease in the rumen CP degradation characterized by lower levels of protein breakdown products (i.e., N-NH_3_, iso-butyrate and iso-valerate) was noted from day 4 to day 21 across treatments. Moreover, the N-NH_3_ values were always below the theoretical threshold (50 mg/L) which can limit the microbial protein synthesis in the rumen (75), indicating that the availability of rumen degradable N was a limiting factor in our experimental conditions. Despite this limitation, the RFF and RFC animals had 2.8 and 6.6 times higher rumen N-NH_3_ concentrations than CTL kids at 4 and 21 days after the dietary shift, respectively, implied a higher N availability for the microbes which could ease the transition to the forage diet. It is known that rumen protozoa can influence the rumen N metabolism at a number of different levels with conflicting effects. Their intense proteolytic activity can explain the positive correlations between rumen N-NH_3_ concentration and the protozoal concentration and richness. On one hand, rumen entodiniomorphids possess a vestibulum surrounded by cilia which make them particularly efficient in taking feed particles and proteins suspended in the rumen, being more active in degrading insoluble than soluble N (76). As a result, up to three entodinimorphid genera (i.e., *Entodinium*, *Diplodinium* and *Polyplastron*) were positively correlated with N digestibility leading to higher values (+16.6%) than in the CTL animals. Similar increments in N digestibility were reported when heifers were repeatedly inoculated with bison rumen fluid containing 2.5 times higher protozoal concentration (77). Increased N digestibility represents a positive digestive adaptation when animals are fed low N diets, and could partially explain the higher rumen N-NH_3_ concentration. Newbold et al. (44) found that increased N digestibility in presence of protozoa is often accompanied by a shift in the N partition, leading to higher urinary N excretion. This phenomenon was not observed in our study, possibly because the urea-N recycling through the saliva was enhanced as a compensatory mechanism (78). On the other hand, rumen bacteria represent the main N source for rumen protozoa (67), this process being associated with increased rumen N-NH_3_ levels and lower microbial protein flow to the intestine (−23%) and the EMPS (−21%), making the rumen less efficient (44). Our findings showed that RFF and RFC animals had approximately half the microbial protein flow than the CTL animals, being this difference greater than observed between faunated and defaunated lambs with higher N intake (7, 46). Low microbial protein flow can represent a relevant handicap for growing, pregnant of lactating ruminants given their high N requirements. Bacterial predation by rumen protozoa is proportional to the protozoal concentration and size, moreover entodiniomorphids had higher predatory activity than holotrich protozoa (47) and ultimately higher impact on microbial protein flow (46, 79). Considering the rumen protozoal concentration and the rates of bacterial CP breakdown activity described for the different protozoal groups (47), it was estimated that 7.7 and 25.3% of the CP intake was broken-down by rumen protozoa in animals inoculated with autoclaved or fresh rumen fluid, respectively. Moreover, considering the proportions of each protozoal taxa it was concluded that most of the bacterial CP breakdown was due to *Entodinium* (83–96%), followed by Diplodiniinae (3.5–6.7%), *Dasytricha* (0.4–5.3%), *Isotricha* (0.1–4.6%) and Ophyoscolecinae (0.01–0.5%). These estimations could explain the positive correlation observed between rumen N-NH_3_ concentration and the abundance of most protozoal taxa including entodiniomorphids (*Entodium_sp_LDK-2011, Enoploplastron, Polyplastron* and *Ohryoscolex*) and hototrich (*Isotricha* and *Dasiytricha*). This suggests that all protozoal groups had, to some extent, bacterial breakdown activity. Interestingly, *Entodinium* was the only protozoal taxa with a negative correlation with urinary PD excretion and EMPS, possibly because they represented the most abundant genus in the rumen. These findings suggest that nutritional strategies based on the use of anti-protozoal feed additives should be re-considered to improve N use efficiency in ruminants (80), especially when they are fed low-N diets.

Our study demonstrated that, in addition to protozoa, the bacterial community was also associated with the rumen N metabolism profile and feed utilization, whereas the impact of methanogens and anaerobic fungi was negligible. In particular, the rumen N-NH_3_ concentration was positively correlated with several taxa including Verrucomicrobiota and Erysipelatoclostridiaceae, *Selenomonas*, *Veillonellaceae_UCG-001* and *vadinBE97*, and negative correlated with *Desulfobacterota* and *Christensenellaceae_R-7*. Although the impact of these bacterial taxa on the rumen N metabolism is largely unknown, it has been described that *Selenomonas ruminantium* can utilize nitrate, urea and amino acids resulting on N-NH_3_ as the main fermentation product (81), as noted in RFF and RFC animals. The *Christensenellaceae*, a recently described family in the phylum *Firmicutes*, is emerging as an important player in human gut health given its inverse correlation to host body mass index and inflammatory bowel disease (82), this microbe being also more abundant in CTL animals. Moreover, our study identified several bacterial taxa such as *Prevotella_ruminicola, Prevotellaceae_Ga6A1_group, Coprococcus, Oscillospira_guilliermondii* and *CAG-352* that had positive correlations with the urinary PD excretion and EMPS, making them candidates for being indicators of efficient N utilization. *Prevotella ruminicola* is one of the few rumen microbes with dipeptidyl peptidase activity facilitating the feed proteolysis and N incorporation into the microbial protein (83). As a result, *Prevotella* and *Oscillospira* have been postulated as indicators of feed efficiency in steers and beef cattle, respectively (53, 58). Similar increases in the levels of *Selenomonas*, *Prevotella* and *Oscillospira* have been described in response to a shift from concentrate feed to grazing diets in sheep (41, 84). A recent study (85) noted that high N efficiency was associated with less diverse rumen bacterial community whereas low N utilization was associated with high abundance of bacteria taxa that promoted greater N excretion through protein degradation in beef cattle. These findings suggest that having a complex rumen microbial community characterized by a high bacterial diversity and presence of rumen protozoa can favor N digestibility but also can limit the microbial protein flow to the intestine (86). Since the low CP content in the forage was a clear dietary limiting factor in this study, the positive effects of having a complex rumen microbiota on the energy metabolism were partially compensated by the negative effects on the N metabolism, resulting on similar animal performances across treatments. These findings underscore the importance of ensuring a sufficient supply of rumen-degradable N when feeding low-quality forages because it promotes a more active and complex rumen microbiota, a phenomenon previously observed in dairy cows (87). This enhanced microbial diversity and activity play ultimately a pivotal role in favoring the degradation of fibrous materials by the rumen microbes.

## Conclusion

This study demonstrated that inoculation of young ruminants with fresh rumen fluid from adult ruminants promoted a greater rumen microbial complexity characterized by higher bacterial and methanogens diversity, as well as the presence of a complex protozoal community, which persisted later in life. This increased rumen microbial complexity represented a competitive advantage when adult animals were fed forage allowing a faster adaptation to the diet and optimized energy metabolism (higher DMI, fiber digestion and VFA production). On the contrary, high rumen microbial complexity had contrasting effects on the N metabolism because it favored the N digestibility but also had a negative impact on the microbial protein flow to the host as a result of increased microbial protein breakdown by the rumen protozoa. These results, suggest that promoting greater rumen microbial diversity is a desirable attribute when animals are fed forages in which the N supply does not represent a limiting factor.

## Data availability statement

The datasets presented in this study can be found in online repositories. The names of the repository/repositories and accession number(s) can be found at: https://www.ebi.ac.uk/ena, PRJEB63607.

## Ethics statement

The animal study was approved by animal procedures were conducted in accordance to the National guidelines (RD53/2013) and were approved by the Ethical Committee for Animal Research (EEZ-CSIC, 09/03/2017). The study was conducted in accordance with the local legislation and institutional requirements.

## Author contributions

AB: Conceptualization, Data curation, Formal analysis, Investigation, Methodology, Resources, Software, Validation, Writing – original draft. JP-H: Methodology, Resources, Writing – review & editing. EJ: Methodology, Resources, Writing – review & editing. DY-R: Conceptualization, Funding acquisition, Supervision, Validation, Writing – review & editing.
