# Supplementary material for: Enhancing rumen microbial diversity and its impact on energy and protein metabolism in forage-fed goats
Source: Front Vet Sci. 2023 Dec 21;10:1272835. doi: 10.3389/fvets.2023.1272835 (PMC10764530; doi:10.3389/fvets.2023.1272835)
Supplement: Supplementary file 1 [file Table_1.DOCX]

Supplementary material

**Enhancing Rumen Microbial Diversity and its Impact on Energy and Protein Metabolism in forage-fed goats**

Alejandro Belanche^1,2*^, Juan Manuel Palma-Hidalgo^1^, Elisabeth Jiménez^1^, David R. Yáñez-Ruiz^1^

^1^ Estación Experimental del Zaidín (CSIC), Granada (Spain)

^2^ Department of Animal Production and Food Sciences, University of Zaragoza, Zaragoza (Spain)

Correspondence: belanche@unizar.es

**Supplementary Table S1.** Primers used for quantitative PCR and Next Generation Sequencing.

| Target | Ref. | Name Primer | Forward Primer | Name | Reverse Primer | Amplicon (bp) |
| --- | --- | --- | --- | --- | --- | --- |
| Quantitative PCR |  |  |  |  |  |  |
| Total bacteria | [1] | 1048F | GTGSTGCAYGGYTGTCGTCA | 1175R | ACGTCRTCCMCACCTTCCTC | 150 |
| Methanogens | [2] | qmcrA-F | TTCGGTGGATCDCARAGRGC | qmcrA-R | GBARGTCGWAWCCGTAGAATCC | 140 |
| Protozoa | [3] | P-SSU-316f | GCTTTCGWTGGTAGTGTATT | PS-SU-539r | CTTGCCCTCYAATCGTWCT | 223 |
| Anaerobic fungi | [4] | qPCR fungi-F | GAGGAAGTAAAAGTCGTAACAAGGTTTC | qPCR fungi-R | CAAATTCACAAAGGGTAGGATGATT | 120 |
| Sequencing |  |  |  |  |  |  |
| Bacteria | [5] | V3_F357 | CCTACGGGAGGCAGCAG | V5_926 | CCGTCAATTCMTTTRAGT | 570 |
| Methanogens | [6] | Arch349F | GYGCASCAGKCGMGAAW | Arch806R | GGACTACVSGGGTATCTAAT | 457 |
| Protozoa | [7] | F566Euk | CAGCAGCCGCGGTAATTCC | R1200Euk | CCCGTGTTGAGTCAAATTAAGC | 660+var. |
| Anaerobic fungi | [8] | ITS3 | GCATCGATGAAGAACGCAGC | ITS4 | TCCTCCGCTTATTGATATGC | 356+var. |

**Supplementary Table S2.** Rumen fermentation and microbial composition of the inocula.

| **Inoculum^1^** | **AUT** | **RFF** | **RFC** | **s.e.d.** | **P-value** |
| --- | --- | --- | --- | --- | --- |
| Fermentation products | | |  |  |  |
| pH | 6.11^b^ | 6.38^a^ | 5.79^c^ | 0.095 | 0.002 |
| Lactate (mM) | 1.86^a^ | 0.55^b^ | 0.85^b^ | 0.328 | 0.017 |
| Ammonia-N (mg/dL) | 8.64 | 7.27 | 10.0 | 1.095 | 0.117 |
| Total VFA (mM) | 120^ab^ | 103^b^ | 134^a^ | 9.990 | 0.055 |
| Acetate (%) | 63.0^b^ | 70.1^a^ | 55.5^c^ | 1.339 | <0.001 |
| Propionate (%) | 23.6^b^ | 18.2^c^ | 29.0^a^ | 1.711 | <0.001 |
| Butyrate (%) | 10.3^b^ | 9.49^c^ | 11.8^a^ | 0.746 | 0.004 |
| Bacterial community | |  |  |  |  |
| Concentration (log10 copies/l) | | 9.96 | 11.8 | 0.884 | 0.081 |
| Richness |  | 502 | 396 | 30.30 | 0.025 |
| Shannon index | | 4.50 | 4.21 | 0.170 | 0.154 |
| Abundance (%) | |  |  |  |  |
| p_Actinobacteriota | | 0 | 0.26 | 0.150 | 0.158 |
| p_Bacteroidota | | 71.5 | 59.1 | 3.060 | 0.015 |
| p_Cyanobacteria | | 0.66 | 0.15 | 0.118 | 0.012 |
| p_Elusimicrobiota | | 0.31 | 0.21 | 0.165 | 0.577 |
| p_Fibrobacterota | | 0.70 | 1.60 | 0.811 | 0.326 |
| p_Firmicutes | | 24.2 | 34.6 | 2.990 | 0.025 |
| p_Proteobacteria | | 2.06 | 0.83 | 0.669 | 0.140 |
| p_Spirochaetota | | 0.21 | 2.31 | 0.930 | 0.087 |
| p_Synergistota | | 0.09 | 0.10 | 0.048 | 0.812 |
| p_Verrucomicrobiota | | 0.07 | 0.04 | 0.026 | 0.221 |
| Firmicutes / Bacteroidota | | 0.34 | 0.59 | 0.070 | 0.023 |
| Methanogens community | | |  |  |  |
| Concentration (log10 copies/ml) | | 6.32 | 7.65 | 1.213 | 0.314 |
| Richness |  | 20.5 | 14.5 | 1.683 | 0.025 |
| Shannon index | | 1.85 | 1.75 | 0.240 | 0.688 |
| Abundance (%) | |  |  |  |  |
| f_Methanobacteriaceae | | 35.8 | 55.2 | 33.30 | 0.043 |
| f_Methanomassiliicoccaceae | | 62.6 | 44.8 | 38.00 | 0.079 |
| f_Methanomicrobiaceae | | 1.61 | 0 | 4.620 | 0.158 |
| *g_Methanobrevibacter* | | 28.7 | 55.2 | 23.10 | 0.005 |
| *g_Methanosphaera* | | 7.12 | 0 | 15.88 | 0.089 |
| Protozoal community | |  |  |  |  |
| Concentration (log10 copies/ml) | | 6.6 | 8.7 | 0.493 | 0.005 |
| Richness |  | 25.0 | 27.0 | 2.890 | 0.527 |
| Shannon index | | 2.24 | 2.46 | 0.187 | 0.297 |
| Abundance (%) | |  |  |  |  |
| *g_Entodinium* | | 48.8 | 49.3 | 0.102 | 0.863 |
| *g_Ophryoscolex* | | 3.19 | 9.02 | 0.737 | 0.126 |
| *g_Diplodinium* | | 0.01 | 0.01 | 0.123 | 0.999 |
| *g_Polyplastron* | | 0.66 | 2.84 | 0.548 | 0.087 |
| *g_Enoploplastron* | | 0.83 | 0 | 0.101 | <0.001 |
| *g_Isotricha* |  | 18.6 | 22.7 | 0.215 | 0.969 |
| *g_Dasytricha* |  | 25.5 | 9.4 | 0.046 | <0.001 |
| *g_*Unclassified | | 2.31 | 6.7 | 0.481 | 0.121 |
| Anaerobic fungal community | | |  |  |  |
| Concentration (log10 copies/ml) | | 6.76 | 7.55 | 0.125 | <0.001 |
| Richness |  | 12.0 | 15.0 | 2.380 | 0.276 |
| Shannon index | | 1.32 | 1.63 | 0.527 | 0.501 |
| Abundance (%) | |  |  |  |  |
| *g_Caecomyces* | | 63.6 | 9.22 | 0.655 | 0.064 |
| *g_Piromyces* |  | 0.0 | 50.0 | 0.006 | <0.001 |
| *g_Neocallimastigaceae* | | 36.4 | 40.8 | 0.107 | 0.621 |

Treatments: Autoclaved rumen fluid (AUT), fresh rumen fluid from adult goats adapted to forage-rich (RFF) or concentrate-rich diet (RFC). Table based on a previous publication (9).

**Supplementary Table S3.** Spearman correlations (*ρ*>0.35, *P*<0.01) between the rumen microbes and productive data.

**References**

1. Maeda H, Fujimoto C, Haruki Y, et al. Quantitative real-time PCR using TaqMan and SYBR Green for *Actinobacillus actinomycetemcomitans , Porphyromonas gingivalis , Prevotella intermedia* , tetQ gene and total bacteria. *FEMS Immunol Med Microbiol* 2003;39:81–86.
2. Denman, S.E., N. Tomkins, C.S. McSweeney, *Quantitation and diversity analysis of ruminal methanogenic populations in response to the antimethanogenic compound bromochloromethane.* Fems Microbiol Ecol 2007;**62**:313-22.

3. Sylvester JT, Karnati SKR, Yu ZT et al. Development of an assay to quantify rumen ciliate protozoal biomass in cows using real-time PCR. *J Nutr* 2004;**134**: 3378-84.

4. Denman SE, McSweeney CS. Development of a real-time PCR assay for monitoring anaerobic fungal and cellulolytic bacterial populations within the rumen. *FEMS Microbiol Ecol* 2006;**58**: 572-82.

5. Sim K, Cox MJ, Wopereis H, et al. Improved detection of bifidobacteria with optimised 16S rRNA-gene based pyrosequencing. *PloS one* 2012;**7**:e32543.

6. Gantner S, Andersson AF, Alonso-Sáez L, et al. Novel primers for 16S rRNA-based archaeal community analyses in environmental samples. *J Microbiol Meth* 2011;**84**: 12-18.

7. Hadziavdic K, Lekang K, Lanzen A, et al. Characterization of the 18S rRNA gene for designing universal eukaryote specific primers. *PloS one* 2014;**9**: e87624.

8. De Beeck MO, Lievens B, Busschaert P, et al. Comparison and validation of some ITS primer pairs useful for fungal metabarcoding studies. *PloS one* 2014;**9**: e97629.

9. Belanche A, Palma-Hidalgo JM, Nejjam I, Jiménez E, Martín-García AI, Yáñez-Ruiz DR. Inoculation with rumen fluid in early life as a strategy to optimize the weaning process in intensive dairy goat systems. *J Dairy Sci*. 2020;103(6):5047–60.
